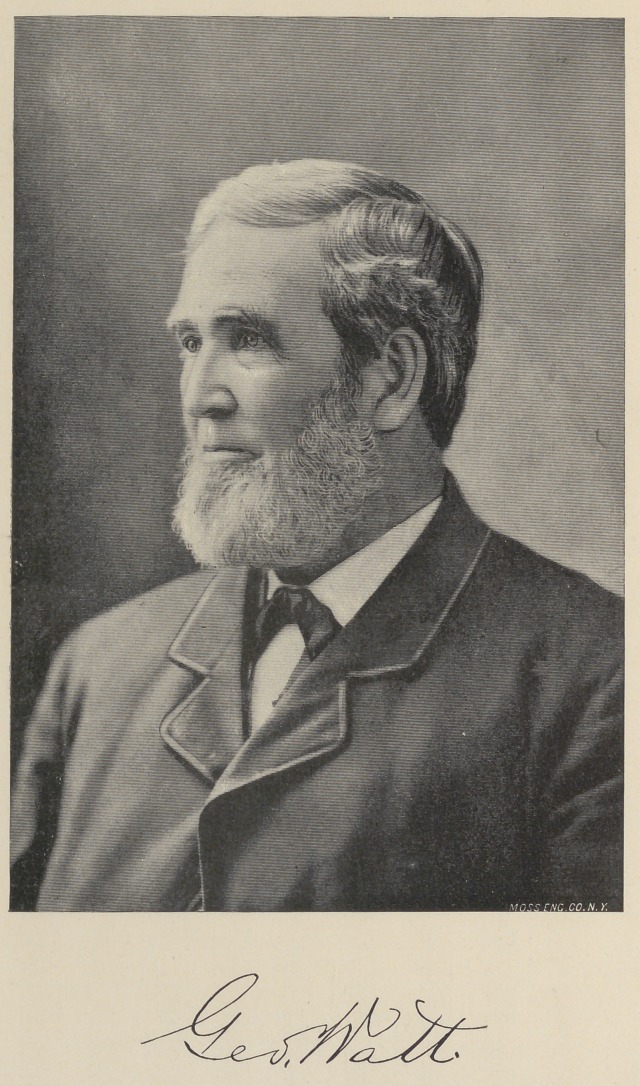# Biographical Sketch of Dr. George Watt

**Published:** 1893-05

**Authors:** 


### THE DENTAL REGISTER.

VOL. XLVII.] MAY, 1893. [No. 5.

### COMMUNICATIONS.

### Biographical Sketch of Dr. George Watt.

Doctor George Watt was born on March 14th, 1820, on a farm eight miles east of Xenia, Greene county, O. His father, Hugh Watt, was born in the north of Ireland, of Scotch parentage; his mother was a native of western Pennsylvania, and was of Scotch parentage also. This portion of Greene county, at the time this home was established, was comparatively new country; many of the animals of the native forest were still there. Dr. Watt remembers seeing wild deer, wolves, bears, catamounts, wild turkeys, foxes, etc., on the land that then constituted their farm.

He first entered a country school at seven years of age. Schools at that time were very primitive and were generally continued only for three or four months during the year. He remained at the family home till October, 1835, when he left, and went to Adams county, O., to enter a boys' academy, established and conducted by Rev. Wm. Taylor. He entered upon his course, agreeing to pay his way, including boarding and tuition, by services such as he could render on the farm, and in connection with the school. He pursued his studies with great industry and made rapid progress, notwithstanding he devoted about one-third of his time to work upon the farm.

This kind of life, away from home and its kindly influences, was a new experience to him; he found an absence of the home surroundings and the sympathy and affection he had so fully enjoyed in his father's house. The treatment he received made the greater impression upon him from the fact that he had not a strong constitution and was often sick. Unfortunately his feeble condition was sometimes regarded as feigned, and because of this he was often-times taxed much beyond his real strength. He had several severe illnesses, and in two or three instances he was supposed to be past recovery. After remaining in the academy two years he engaged in teaching, but a few miles distant from the academy. He was thus engaged for three or four years; a part of this time, however in the neighborhood of his father's home. In 1840 he entered a college at Ripley, Brown county, O., and remained about a year, leaving there in 1841, returned to Greene county, and there engaged in teaching and the study of medicine, in which he had the late Dr. Samual Martin, of Xenia, for his preceptor, with whom he not only had surperior instruction in medical science and practice, but also had there inculcated, in a receptive mind, the views of the dignity and importance of medical science and practice that have ever characterized him, throughout his life.

He practiced with and for his preceptor about one year; after which he removed to Bentonville, a small town in Fayette county, Indiana, where he soon established an excellent practice, especially for a new country. Not being satisfied with his attainments in medical science, he entered the Medical College of Ohio in 1846, and graduated in March, 1848.

April 17th, 1845, he was married to Miss Sarah Jane McConnell, who was a native of Greene county, Ohio, and had always resided with her father's family near Xenia. Both she as well as the subject of this sketch were the youngest of their respective families. The two families here referred to were for a long time neighbors and intimate friends, indeed they journeyed together in coming to Ohio.

After the completion of his medical course, he continued the practice of his profession in Indiana about one year. Near the close of 1848 Mrs. Watt was poisoned by taking arsenic which had been concealed in an apple, intended as was supposed, for the Doctor himself; the case came well-nigh being fatal; recovery from this was slow, and the result, as to recovery, was for a long time in doubt. This condition of affairs made it necessary for the Doctor to leave his practice and return to the former home of Mrs. Watt. This change of matters brought him to the practice of medicine in Xenia, in which he remained till the spring of 1850, when he removed to Kenton, Ohio, where he continued in practice about two years. About this time his love for and inclination to special scientific pursuits began to be rapidly developed, as was shown by various investigations in which he engaged.

Early in the year 1852, he entered upon the study of dentistry, he was well prepared for such a course of study, by his medical knowledge. After some time devoted to this specialty, he formed a co-partnership with J. Taft, of Xenia, Ohio, for the practice of dentistry which continued for many years. One branch of science to which Dr. Watt had given much attention, during and after his medical course, was chemistry. Some idea of his proficiency in this direction will be apparent when it is remembered that he had an unusual aptitude in and liking for this branch, and also that he had for his preceptor that noted chemist and philosopher, the late lamented Professor John Locke, who was for many years a teacher in the Medical College of Ohio. In 1853 he prepared and delivered a course of lectures on chemistry in the Ohio College of Dental Surgery. And though Dr. Elijah Slack, M.D., LL.D., had delivered a course of lectures on chemistry in the same institution for one or two years before, yet this effort of Dr. Watt was the first attempt to adapt a course of lectures on chemistry to the need of the dental students, and he was the first to prepare and deliver such a course. All work in this line done before him was simply such as was given to medical students. During the time in which this course was delivered, he was not only a teacher hewing out a new course for himself, but he was a member of the class he taught, so that he was in the double capacity of a teacher and pupil.

He graduated with his class, receiving the degree of Doctor of Dental Surgery at the close of the term of 1854.

And though he was a graduate of medicine with quite an extensive and varied experience in its practice, and had given more than one year's time to the study of dentistry, and was a teacher of acknowledged ability, yet, at the time of his graduation, he passed the same examination as the other members of his class. Immediately after this he resumed the practice of dentistry with Dr. Taft. So well recognized was his ability as a teacher, that he was not long permitted to enjoy the quiet and comfort of a town and country practice, for in the spring of 1855 he was elected Professor of Chemistry and Metallurgy in the Ohio College of Dental Surgery, which position he occupied for several years, and the result achieved by him during this time in the development of the application of chemistry to dental science and art, has never been excelled if equalled in the same length of time by any teacher in any other branch. He was the pioneer in directing the attention of the profession to chemistry, as one of the chief factors in dental art and science.

He became a member of the Mississippi Valley Dental Society in 1852. In 1854 a prize of one hundred dollars was offered for the best popular essay on Dental Surgery.

Dr. Watt was a competitor for this prize, and after a thorough examination of all the papers presented, it was unanimously awarded to him. He was for several years secretary of the body, and after many years of service in almost every other capacity, he was elected its president.

In 1856 he was a member of the American Dental Convention (the largest dental body then in existence) held at Hope Chapel in New York.

At the meeting he read a paper on "Topical Remedies," which was extensively published and elicited much attention and discussion, and at the time of its reading drew forth quite a varied discussion. The article is contained in Watt's "Chemical Essays," and is also published in the tenth volume of the *Dental Register*.

He with Dr. Taft, became the owner and editor of the *Dental Register of the West*. This relation was maintained for many years and was only relinquished when Dr. Watt's failing health rendered it imperative. But he has always, and even after this severance, been a liberal contributor to the literature of the profession.

He with Doctors Taft and Hamill, established an office for dental practice in Cincinnati, in 1855. This relationship was only sustained for two or three years, the sudden death of Dr. Watt's father made it necessary for him to remove to Xenia, Ohio, when he entered the office and continued the practice that had been established many years before. About this time he made some experiments in micro-photography that were in advance of anything that had been accomplished, and perhaps even attempted up to that time.

In the autumn of 1860, in the time of the great trial of our nation, he promptly tendered his services so far as possible for its rescue; however, he was not accepted until May, 1864, when he was made surgeon of the 154th regiment of Ohio Volunteer Infantry.

His efficiency in that position will be well understood, when it is stated that the sanitary record of his charge was better than that of any other Ohio regiment.

He was mustered out September, 1864, after being disabled by an injury to the spine, having been crushed by a falling wagon, which resulted in locomotor ataxia.

After his return from the army he entered into practice, as his feeble health would permit.

In the autumn of 1865 he formed a partnership with Dr. N. W. Williams, after which they conducted a practice in Xenia for about one year, and they established a branch office in Cincinnati of which Dr. Watt took charge.

In 1868 the firm of Watt & Williams purchased the dental depot of J. S. Walters & Co., of Cincinnati. This business they conducted successfully in addition to their dental practice for about three years, when they sold the depot to Spencer & Moore. During this last residence in Cincinnati, his health and strength had gradually become much impaired, and after disposing of his commercial enterprise and resigning his position in the college, where he had been teaching for three years, he closed his city office, and on September 30th, 1871, removed to Xenia, where he hoped to have respite from active labor, but his active brain would not permit much rest.

In the spring of 1872 the firm of Watt & Williams was dissolved, Dr. Williams going to Europe. Dr. Watt found himself unable to carry on the large practice then on his hands, and so he formed a partnership with Dr. D G. French, which continued a little less than one year, after which he formed a partnership with Dr. E. G Betty, of Cincinnati. This relation was also continued about one year, after which a partnership was formed with Dr. W. H. Sillito, and the business was conducted some two or three years by the firm of Watt & Sillito.

During all of this time his health was becoming more feeble and his strength at times seemed well-nigh gone, and it was deemed best that he should retire and no longer attempt the active duties of practice. But he was not content to be idle. In 1881 he took the editorship of the *Ohio Journal of Dental Science*, a monthly journal of about fifty pages, and what it has been from the beginning to the present we need hardly attempt to describe here; sufficient to say that it occupies an enviable position with its compeers; its pages are always freighted with matter of interest and profit, and not the least of this, by any means, is from the editor. The editorials are always rich in thought, and clearly characteristic of the man even in his best days.

During the time of this work he has been a marvel to all who knew of his condition. Clearly, his brain has not shared in the debility which has seemed to pervade all the rest of his organism.

In 1867 he published a volume entitled "Watt's Chemical Essays," in which are published the principal papers which he has written on dental chemistry.

Quite a number of these papers have been published in various journals, not only of the dental but the medical profession as well, and even in some of the leading newspapers of the country. Unfortunately, the syllabus of his series of lectures on chemistry has never been published.

He has occupied a number of positions of prominence in addition to those already named. He was Vice-President of the American Dental Convention; he was elected President of the American Dental Association on the same day he became a member, the only instance of the kind on record. He was President of the Ohio State Dental Society the first two years of its existence; was twice President of the Mad River Dental Society. In every responsible position he ever occupied, the duties devolving on him were discharged faithfully and efficiently.

Dr. Watt was one of the most easy, fluent and accurate writers in the dental profession; his literary taste was formed upon a high model, and was all that could be expected of one of the highest literary and classical culture. In his case it was rather inborn than acquired, though as some explanation, it may be said that his reading in early life was confined to the purest and best of English literature."

Dr. Watt's last severe illness began about December, 1892, since which time he has gradually failed. The last dental meeting he attended was the Seventh District Society of Ohio, at Xenia, May, 1891. His interest in society work never flagged so long as he was able to attend. For the last year and a half of his life he wrote very little, his mind somewhat failing in concentration. For the last few months he sank gradually away. He possessed a remarkable constitution. During the last twenty-five years of his life he was subject to very severe attacks of disease, many of which would have proved fatal with almost any other person. His power of discrimination of disease in its various phases was very great indeed, and even in his own case he recognized his own condition so long as his mind was clear, seemingly better than any other physician could do. He was always able to describe to his attending physician his symptoms most clearly, and usually was able to point out the appropriate treatment. To this, no doubt, was due the fact that he passsd through so many attacks of severe disease and retained a good degree of health; but by-and-by his great strength became exhausted, and he was compelled to yield to the destroyer of all living.

---

Breathing through the nose is the only proper way to sleep. If you awake in the night and find your mouth open, get up and shut it.

## Figures and Tables

**Figure f1:**